# Variation of outcome reporting in studies of interventions for heavy menstrual bleeding: a systematic review

**DOI:** 10.52054/FVVO.14.3.029

**Published:** 2022-09-30

**Authors:** N.A.M. Cooper, R Papadantonaki, S Yorke, K.S. Khan

**Affiliations:** Women’s Health Research Unit, Queen Mary University of London, UK; Integrated Academic Training programme, London Specialty School of Obstetrics and Gynaecology, London, UK; London and Kent, Surrey and Sussex Foundation Programme, UK; Department of preventative medicine and public health, University of Granada, Spain; Centro de Investigación Biomédica en Red (CIBER) Epidemiología y Salud Pública, Instituto de Salud Carlos III, Madrid, Spain

**Keywords:** Core outcome set, heavy menstrual bleeding, outcome variation, methodology

## Abstract

**Background:**

Heavy menstrual bleeding (HMB) detrimentally effects women. It is important to be able to compare treatments and synthesise data to understand which interventions are most beneficial, however, when there is variation in outcome reporting, this is difficult.

**Objectives:**

To identify variation in reported outcomes in clinical studies of interventions for HMB.

**Materials and methods:**

Searches were performed in medical databases and trial registries, using the terms ‘heavy menstrual bleeding’, menorrhagia*, hypermenorrhoea*, HMB, “heavy period „period“, effective*, therapy*, treatment, intervention, manage* and associated MeSH terms. Two authors independently reviewed and selected citations according to pre-defined selection criteria, including both randomised and observational studies. The following data were extracted- study characteristics, methodology and quality, and all reported outcomes. Analysis considered the frequency of reporting.

**Results:**

There were 14 individual primary outcomes, however reporting was varied, resulting in 45 specific primary outcomes. There were 165 specific secondary outcomes. The most reported outcomes were menstrual blood loss and adverse events.

**Conclusions:**

A core outcome set (COS) would reduce the evident variation in reporting of outcomes in studies of HMB, allowing more complete combination and comparison of study results and preventing reporting bias.

**What is new?:**

This in-depth review of past research into heavy menstrual bleeding shows that there is the need for a core outcome set for heavy menstrual bleeding.

## Introduction

Heavy menstrual bleeding (HMB) is one of the most common reasons for referral to secondary care and affects up to 1 in 5 women of reproductive age (Coulter et al., 1989). It is a common condition with a large impact on women’s physical, psychological and social wellbeing (Coulter et al., 1994; Jones et al., 2002; Clark et al., 2002) as well as an economic impact due to time taken away from employment and cost to the healthcare systems (Coulter et al., 1995; Coulter et al., 1988). There are many different treatments for HMB including hormonal, medical and surgical interventions. Although these treatments have been widely explored in clinical trials, these trials do not all report their results using the same outcomes, preventing comprehensive data synthesis, and reducing the impact on clinical guidance.

Although checklists exist for the reporting of clinical trials, ‘core outcome sets’ (COS) differ because they are a disease-specific agreed set of outcomes that are established as a reporting standard minimum for all relevant clinical trials. The aim of a COS is to ensure that studies of a condition all report the same, valid outcomes which will allow future data synthesis for development of clinical guidelines and will also prevent selective outcome reporting. Ultimately, this will mean that all studies which are conducted into a condition will produce results that are not only useful for interpretation of that trial but can also contribute to meta-analyses and the overall assessment of interventions. This will make results more valuable, more meaningful in comparisons and more likely to influence improvements in policy and practice.

The aim of this systematic review was to identify outcomes that have previously been used in studies of interventions for HMB and examine the variation in reporting. This is the first stage in the development of a COS for HMB for use in future trials. A previous review explored the primary outcomes from randomised controlled trials of HMB (Bongers et al., 2017), however as only randomised controlled trials (RCTs) were used, other important outcomes were potentially excluded. For development of a COS, it is important that all potential outcomes are considered, therefore this review examines all outcomes and is not restricted to RCTs.

## Methods

We performed a systematic review in line with current recommendations (Higgins, 2021) as part of the development of a COS for HMB. We prospectively registered the review with PROSPERO (reference: CRD42018093239) and the COS study with the COMET (Core Outcome Measures in Effectiveness Trials) Initiative (project reference number 789). This work was funded by a starter grant from the Academy of Medical Sciences.

### Literature reviews and trial registry searches

We conducted a comprehensive literature search to identify studies of heavy menstrual bleeding. Searches were performed in Medline (1946 to 23rd January 2019), EMBASE (1974 to 23rd January 2019), CINAHL (1981 to 23rd January 2019) and AMED (1985 to 23rd January 2019) to identify relevant trials and systematic reviews. Search terms used included ‘heavy menstrual bleeding’, menorrhag*, hypermenorrh*, HMB, “heavy period“, effective*, therapy*, treatment, intervention, manage* and associated MeSH terms. Boolean operators AND or OR were used as appropriate and no language restrictions were applied. All search strategies are presented in Appendix I. In addition, clinical trial registers (CENTRAL, EU clinical trials register, clinicaltrials.gov, International Standard Randomized Controlled Trial Number (ISRCTN) register etc.) were searched to identify trials not published or not yet completed and which had not been identified by the medical database searches. The search terms were ‘heavy menstrual bleeding’ and menorrhagia.

## Study selection

Two authors (NAMC and RP or SY) independently reviewed the titles and abstracts from the electronic literature searches and selected citations if they seemed to fulfil the selection criteria which were as follows:

### Population:

studies of patients with HMB. Studies that were of mixed (e.g., also involved patients with intermenstrual or postmenopausal bleeding) or specific (e.g., patients with coagulopathy or intrauterine contraceptive device induced HMB) populations were excluded. If studies were of patients with fibroids or adenomyosis, the primary outcome had to be related to menstrual blood loss (MBL) or quality of life rather than to other associated symptoms that are specific to these conditions e.g., shrinkage of fibroids, pain.

### Intervention:

any intervention for HMB whether that be medical, surgical, or other.

### Study type:

randomised controlled trials, observational studies with ≥ 100 participants and systematic reviews and meta- analyses (for the purpose of cross-referencing the included studies and identifying studies not identified by our searches). Case reports were excluded.

When duplicate data were published, we included the primary study and excluded any later follow-up studies. In the case of systematic reviews which had undergone updates, we used the most recent version. Studies selected by both reviewers were included. Any disagreements about study eligibility were resolved by consensus. The complete manuscripts of selected citations were then reviewed in full to determine inclusion or exclusion and this list was then cross-checked against the trial registry searches and the lists of studies included in selected systematic reviews to identify additional relevant studies.

## Data extraction and analyses

Data were extracted by one author (NAMC) regarding, study characteristics and methodology, study and outcome reporting quality and all reported outcomes. A second author extracted data from 10% of the included studies to confirm accurate data extraction. Outcomes were considered as the primary study outcome if they were identified in the study as so, or if they were used in the power calculation for the study. If neither of these were evident but a ‘main’ outcome was stated this was also considered as a primary outcome. All other outcomes were secondary outcomes. Outcomes specific to fibroids were excluded (e.g., change in fibroid volume). The frequency and variation of all reported outcomes were identified along with the variation of outcome reporting tools and reporting time-points for primary outcomes. Data were extracted from all included RCTs. The observational studies were put in chronological order, and data were extracted from ten studies at a time, starting with the most recent, until no new outcomes were identified. Data regarding planned outcomes were extracted from the trial registry citations.

## Risk of bias assessment

The quality of randomised studies was assessed using the Cochrane risk-of-bias tool for randomized trials (RoB 2) (Sterne et al., 2019). Non randomised studies were assessed using the Newcastle Ottawa quality assessment scale for non-randomised studies (Wells GA). To differentiate between cohort and case series, we used characteristics as described by Esene et al. (2014).

## Results

**Figure 1:**
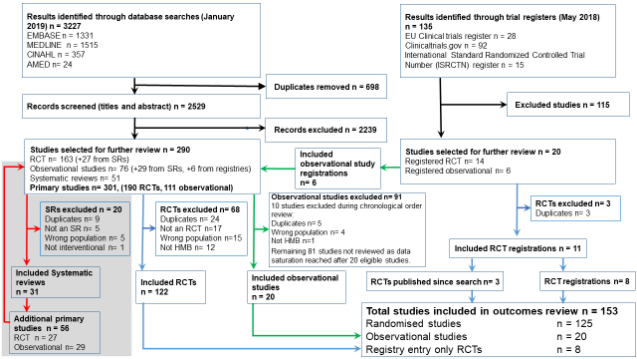
A PRISMA flow diagram detailing study selection for the systematic review of variation of outcome reporting in studies of interventions for heavy menstrual bleeding.

Our medical database searches identified 3227 citations. Once duplicates were removed 2529 citations remained. 290 studies were selected for further review- 163 RCTs, 76 observational studies and 51 systematic reviews. (SRs). The SR full texts were evaluated and 31 met our inclusion criteria. These 31 reviews included a total of 451 studies. We cross checked this list against our citations and found 56 (27 RCTs and 29 observational studies) additional studies which were added to our list for full text evaluation, totalling 190 RCTs and 105 observational studies (See Figure 1). We were unable to obtain the full text manuscripts for 6 studies and so extracted data from the abstract only (Romer, 1998; Hoshiai et al., 2017; Ergun et al., 2012) or, if the study had been included in a systematic review (Buyru et al., 1995; Römer et al., 1996; Jaisamrarn et al., 2006) we identified and used relevant data from that review. Following review of the RCT full texts, 68 were excluded (see Appendix II), leaving 122 RCTs for inclusion in the review.

We identified 135 additional potentially relevant studies from our trial registry searches and after two reviewers (NAMC and RP) reviewed the titles separately, 20 (14 RCTs and 6 observational studies) were evaluated further using the full registry entry. Two RCT entries (Cooper, 2006; AbbVie, 2011) were excluded as they were already included in our review in their full text form (Sambrook et al., 2009; Archer et al., 2017). Four RCT entries ( (Famuyide, 2010; Bayer, 2012; AbbVie, 2013; Owens, 2013) had been published during the course of this work, so included in their full text form (Carr et al., 2018; Famuyide et al., 2017; Yu et al., 2018). Eight RCT registrations remained for inclusion. The observational study registrations were added to the list of observational studies to be analysed.

The 111 observational studies were put in reverse chronological order with the most recent study first. We initially evaluated the eligibility of each study and when we reached 10 eligible studies, we extracted data from them. We continued to analyse 10 eligible studies at a time until we identified no new outcomes. Data saturation was reached after data were extracted from the first 20 eligible studies (30 studies evaluated, 10 excluded- 5 duplicate publications (Chudnoff et al., 2012; Cash et al., 2012; Banks et al., 2012; Muse et al., 2011; Jensen et al., 2013), 4 with mixed populations (Hachmann-Nielsen and Rudnicki, 2012; Pisco et al., 2009; Krogh et al., 2009; Chapa et al., 2009), and 1 which was not assessing HMB (Bansi- Matharu et al., 2013).

The total number of studies included in the review was 153, comprising 125 RCTs, 20 observational studies and 8 trial registry entries. (See Appendices III and IV).

## Study characteristics

The included studies were published between 1965 and 2019. Studies were conducted in 52 countries (Africa n=2, Asia n= 13, Australasia and Oceania n= 2, Europe n= 28, North America n=5, South America n=2), 2 of which are considered low- income countries, 20, middle income countries and 30 high income countries. The population size in the studies ranged from 16 to 723 participants (studies with fewer than 100 were only included if they were RCTs). Further detail is reported in Appendices III and IV.

## Study quality

Of the 125 RCT’s, 82 were assessed to have a high risk of bias and 36 to have ‘some concerns’ regarding the risk of bias. It was not possible to assess seven studies as we were only able to access abstracts. The observational studies were similarly at risk of bias, with 5 being at ‘very high risk’ (score 1-3), 10 being at ‘high risk’ (score 4-6), 1 at ‘low risk’ (score 7-9) and 4 unable to be assessed. We did not attempt to assess the quality of the trial registry entries.

We attempted to evaluate the quality of the study outcome reporting using criteria described by Harman et al. (2013).

All study quality assessment data are detailed in appendices V to VIII.

## Interventions evaluated

72/153 (47%) trials evaluated medical interventions (including LNG-IUS) for HMB, 51/153 (33%) evaluated surgical treatments (including UAE) and 30/153 (20%) evaluated medical interventions against surgical ones. Appendix IX gives further details about the different treatments that were evaluated by the included studies.

## Primary Outcomes

58/153 studies (Alborzi et al., 2002; Barrington et al., 2003; Bonduelle et al., 1991; Buyru et al., 1995; Callender at al., 1970; Cameron et al., 1987; Cetin et al., 2009; Chamberlain et al., 1991; Chang et al., 2009; Chimbira et al., 1980; de Souza et al., 2010; Dockeray et al., 1989; Drosdal, 1993; El Makhzangy et al., 2010; Ergun et al., 2012; Fraser et al., 1981; Fraser et al., 1996; Fraser and McCarron, 1991; Gannon et al., 1991; Ghazizadeh et al., 2011; Ghazizadeh et al., 2014; Hall et al., 1987; Higham and Shaw, 1993; Jaisamrarn et al., 2006; Kriplani et al., 2001; Kriplani at al., 2006; Kucuk et al., 2008; Lamb, 1987; Li et al., 2013; Lissak et al., 1999; McClure et al., 1992; Mirzaei et al., 2018; Naafe et al., 2018; Nilsson and Rybo, 1965; Ozdegirmenci et al., 2011; Römer, 1998; Römer et al., 1996; Sørensen et al., 1997; Sowter et al., 1997; van Zon-Rabelink et al., 2003; Vercellini et al., 1998; Vermylen et al., 1968; Vihko et al., 2003; Vilos et al., 2010; Wing et al., 2006; Ylikorkala and Pekonen, 1986; Zhang et al., 2008; Chudnoff et al., 2013; Glasser et al., 2009; Gultekin et al., 2009; Kdous et al., 2008; Lee et al., 2017; Lete, 2008; Muse et al., 2012; Nakayama et al., 2014; Quesnel-García- Benítez et al., 2016; Varma et al., 2010; Vaughan and Byrne, 2012) did not report a primary outcome (see methods for our definition of primary outcome). In the remaining 95 studies (Abbott et al., 2003; Abdel and Shawki, 2006; Hashim et al., 2012; Agarwal et al., 2016; Ambat et al., 2009; Archer et al., 2017; Athanatos et al., 2015; Bhattacharya et al., 1997; Bongers et al., 2004; Bonnar and Sheppard, 1996; Bradley et al., 2016; Brun et al., 2006; Busfield et al., 2006; Carr et al., 2018; Chudnoff et al., 2017; Clark et al., 2011; Cooper et al., 2002; Cooper et al., 2004; Cooper et al., 1999; Cooper et al., 1997; Corson, 2001; Corson et al., 2000; Crosignani et al., 1997a; Crosignani et al., 1997b; Donnez et al., 1997; Donnez et al., 2016; Donnez et al., 2012a; Donnez et al., 2012b; Donnez et al., 2014; Duleba et al., 2003; Dunphy et al., 1998; Dwyer et al., 1993; Edlund et al, 1995; El-Nashar at al., 2009; Endrikat et al., 2009; Erian et al., 1998; Famuyide et al., 2017; Fathima and Sultana, 2012; Freeman et al., 2011; Garza-Leal et al., 2010; Ghazizadeh et al., 2011; Goshtasebi et al., 2015; Grover et al., 1990; Gupta et al., 2013; Hawe et al., 2003; Hazard and Harkins, 2009; Hoshiai et al., 2017; Hurskainen et al., 2001; Irvine et al., 1998; Istre and Trolle, 2001; Jain et al., 2016; Johns and Harris, 2016; Kashefi et al., 2015; Kaunitz et al., 2010; Kiseli et al., 2016; Kriplani et al., 2012; Laberge et al., 2017; Laberge et al., 2015; Lähteenmäki et al., 1998; Lee et al., 2013; Lukes et al., 2010; Mawet et al., 2014; Meyer et al., 1998; Pellicano et al., 2002; Penninx et al., 2016; Penninx et al., 2010; Perino et al., 2004; Preston et al., 1995; Rahi et al., 2016; Reid et al., 2005; Sambrook et al., 2009; Sayed et al., 2011; Sesti et al., 2012; Sesti et al., 2011; Shabaan et al., 2011; Shaw et al., 2007, Shokeir at al., 2013; Shravage et al., 2011; Soysal et al., 2002; Soysal et al., 2001; Srivaths et al., 2015; Tajjamal and Zaman, 2015; van Zon-Rabelink et al., 2004; Vargyas et al., 1987; Volkers et al., 2007; Yu et al., 2018; Zupi et al., 2003; Bayer, 2006; Gompel, 2009; Cooper, 2010; Sharma, 2011; Bhattacharya, 2014; Bayer, 2015; Critchley, 2015; Nazac, 2015), 115 primary outcomes were reported (some studies stated more than one primary outcome). When evaluated, 14 individual primary outcomes were identified however these were reported using 45 different outcome measures (see Table I and Appendix X). ‘Menstrual blood loss’ (MBL) outcomes were reported most (49 studies), followed by ‘amenorrhoea’ (19 studies), ‘treatment success’ (17 studies), ‘satisfaction’ (11 studies) and ‘Quality of life / patient reported outcome measures (PROMS)’ (7 studies). The remaining nine outcomes were each reported three times or fewer. Medical studies and studies looking at a combination of medical and surgical treatments both reported MBL primary outcomes most frequently, 34/54 (63%) and 7/15 (47%) studies respectively. In contrast, surgical studies used a MBL primary outcome just 8/45 (18%) times, with amenorrhoea being the most used outcome in this subgroup, reported 12 times (26%).

**Table I:** Summary of the 14 primary outcomes and the 45 different ways that they were reported.

Primary outcome	Outcome measure	Number of studies using this as a primary outcome
1. Menstrual blood loss	Change from baseline PBLAC	12
	PBLAC end score	12
	Change in measured menstrual blood loss from baseline (ml)	9
	Mean / median measured menstrual blood loss (ml)	8
	Change in number of bleeding days	2
	Resolution of HMB	2
	HMB still present	1
	Proportion of women with a total PBLAC score <10	1
	Change in intensity of bleeding	1
	Change in average number of pads used	1
2. Amenorrhoea	PBLAC score	7
	No definition provided	3
	Ordinal / categorical categories	2
	Measured by alkaline haematin	2
	Defined as no scheduled or unscheduled bleeding/spotting after the end of the initial bleeding episode	2
	No more than 1 day of spotting in 35-day period	1
	No bleeding or bleeding insufficient to require sanitary protection	1
	Assessed by VAS	1
3. Successful treatment	PBLAC <=75	8
	Measured menstrual blood loss < 80ml or >50% reduction from baseline	3
	PBLAC score <75	3
	Measured menstrual blood loss <= 80ml	1
	PLBAC score < 100	1
	Success not defined	1
4. Satisfaction	6-point likert scale	3
	4-point likert scale	3
	Likert scale with the number of categories not stated	2
	5-point likert scale	1
	Yes / No	1
	Short-form 36 (SF-36) *	1
5. QoL / PROMs	Change in Menorrhagia Multi-Attribute Scale (MMAS) score	2
	Change in EuroQol-5D score	2
	Menorrhagia Multi-Attribute Scale (MMAS) score	1
	Change in Paediatric Quality of Life Inventory (PedsQL) score	1
	Ruta Menorrhagia Questionnaire score	1
6. Haemoglobin	Haemoglobin level as mean and SD gr/dl	2
	Change in haemoglobin	1
7. Continuation of Rx	Number of women	2
8. Hysterectomies avoided	Number of women	1
9. Absence of surgical re-intervention	Number of women	1
10. Failed Treatment	Number of women needing repeat treatment or hysterectomy	1
11. Endometrial thickness	Millimetres	1
12. Cost	Cost per QALY gained	1
13. Change in back pain	NS	1
14. Change in abdominal pain	NS	1
Total	45	115

NS- not specified; QoL = Quality of life; PROM= patient reported outcome measure; * SF-36 is not normally used as a measure of satisfaction, but this is what the authors specified. NB. A more extensive summary is available in Table S6.

Of the 45 outcome measures, the most reported were ‘change in pictorial blood loss assessment chart (PBLAC) score’ and ‘PBLAC end score’ (i.e., did not assess change from baseline), which were both reported as a primary outcome 12 times. The next most frequent were ‘change in measured menstrual blood loss’ (used 9 times) ‘mean / median measured blood loss’ (used 8 times), ‘successful treatment defined as a PBLAC score <75’ (used 8 times) and ‘amenorrhoea defined by PBLAC score of zero’ (used 7 times). All other outcome measures were used three times or less. (See Table I).

The time-point within a study when outcomes are reported is another important factor when assessing interventions. Primary outcomes were reported at 14 different time-points, ranging from ‘1 month’ to ‘60 months’ (see Table II). The most used time point was 12 months which was used 47 times; 8 in medical studies, 31 in surgical studies and 8 times in combination studies. The second most frequent time-point was ‘at the end of treatment’ which was used 30 times and was only used by studies of medical interventions. However, the treatment courses varied in length (ranging from 35 days to 4 courses of treatment each lasting 3 months) and so this time-point was not consistent. The most used ‘end of treatment’ time-point was 3 months (also 3 cycles or 12 weeks) which was used 21 times. ‘3 months’ was used as a time-point an additional 10 times, thus overall, ‘3 months’ becomes the second most frequently used time point being used 31 times. ‘6 months’ and ‘24 months’ are the next most frequently used time-points being used 21 and 9 times respectively. As shown in Table II, medical studies favoured ‘3 months’ as a reporting time- point and surgical studies favoured ’12 months’.

**Table II:** Time-points used by the included studies to assess primary outcomes.

Time-point		Number of times studies used this time-point to report a primary outcome			
		All studies	Medical studies	Surgical studies	Medical and surgical studies
12 months		47	8	31	8
6 months		21	5	11	5
3 months *		10	3	5	2
24 months		9	2	6	1
1 month		3	3	0	0
4 months		2	0	1	1
Other^		5	1	4	0
3 months after the end of treatment		3	3	0	0
At the end of treatment		30	30	0	0
	3 cycles / 3 months / 12 weeks	21	21		
	6 cycles / months	3	3		
	2 cycles	2	2		
	4 x 3/12 courses	2	2		
	7 months	1	1		
	35 - 50-day course	1	1		
Total		130	55	58	17

*Also see ‘At the end of treatment- 3 cycles / 3 months / 12 weeks; ^1 x 48 months; 2 x 36 months; 1 x 12 to 60 months; 1 x1.3 to 3.5 years. Some studies used multiple primary outcomes, and some reported at multiple time-points which accounts for the discrepancy between the number of studies included in the review and the number of outcomes and time-points.

Some studies used multiple primary outcomes, and some reported at multiple time-points which accounts for the discrepancy between the number of studies included in the review and the number of outcomes and time-points.

## Secondary outcomes

There were 91 identified secondary outcomes, reported in 343 different ways. Some of these outcomes were the same general outcome but reported as a ‘change from baseline’ as well as a ‘follow-up only’ outcome (e.g., ‘change in severity of dysmenorrhoea’ versus ‘severity of dysmenorrhoea’). Many outcomes were the same outcome but worded differently, or the converse of each other, for example ‘no response to treatment’ and ‘number who still have HMB’. When these similar outcomes were consolidated (see Appendix XI), there were 165 specific secondary outcomes. Each of the identified primary outcomes were also identified as secondary outcomes except for ‘hysterectomies avoided’.

The ten most frequent secondary outcomes are shown in Table III. Overwhelmingly, the most reported was ‘menstrual blood loss’, used 230 times and reported using 16 outcome measures (the most common being PBLAC scores, subjective assessments and measured MBL) – see Appendix XI. The next most common were ‘surgical complications’, ‘quality of life’, ‘haemoglobin value’ and ‘satisfaction’ reported 85, 76, 73 and 66 times respectively. ‘Side effects and adverse events were the 9^th^ and 10^th^ most frequently used outcomes (reported 43 and 42 times). Whilst some studies reported ‘adverse events’ overall, others reported ‘side effects’ or ‘complications’. If these are combined and considered to all be ‘adverse events’ this becomes the second most frequently reported secondary outcome, being used 172 times. Four of the primary outcomes, were found to be amongst the top ten most common secondary outcomes (MBL, quality of life, satisfaction, and haemoglobin level), reinforcing their popularity and likely relevance.

**Table III:** The ten most commonly reported secondary outcomes in the included studies.

Overall outcomes	Number of times reported*
Menstrual blood loss	230
Rate, type and timing of surgical complications	85
Quality of life / PROMs	76
Haemoglobin value	73
Satisfaction with treatment	66
Additional treatment or re-intervention for HMB	56
Duration of menses	55
Length of procedure (start and end point defined)	44
Side effects	43
Adverse events	42

* Some studies reported multiple specific outcomes (as might be expected when exploring secondary outcomes) which may have fallen under one overall outcome more than once, for example a study may have reported ‘measured menstrual blood loss (at study time-point)’, ‘number of women with blood clots’ and ‘change in measured menstrual blood loss’, so will have been counted 3 times in the ‘menstrual blood loss’ overall outcome. Hence the large number of outcomes compared to the number of studies.

60 of the 91 outcomes were reported 10 times or fewer, with 41 of these being reported 5 times or fewer. See Appendix XI.

When the primary and secondary outcomes are considered with their reporting measures and consolidated, we identified 166 outcomes overall.

## Discussion

### Main findings

We found a wide variation in outcome reporting for studies of interventions for HMB as might well be expected for a condition that can be managed in multiple ways and can be caused by several underlying pathologies. This variation occurred across types of outcomes as well as how and when they were reported. Menstrual blood loss was the most reported primary and secondary outcome and was particularly favoured by studies which involved a medical intervention. In contrast, amenorrhoea was favoured as a primary outcome by surgical studies.

### Strengths and Limitations

This systematic review is a comprehensive look at medical literature regarding HMB over the last 50 years. Observational work has been used as well as randomised studies to allow identification of alternative outcomes. We also identified outcomes that were being used in planned and ongoing studies by examining study registers. Strict methodology was used to conduct the systematic review.

We explored secondary outcomes as well as primary outcomes to ensure full overview of study reporting and unlike previous work, we looked at reporting methods and timing. We used a broad definition of primary outcome to account for advances in study methodology and reporting over the years and to ensure that relevant studies were included. Studies with no primary outcome were not excluded from our review to ensure that we captured as many outcomes as possible for reporting HMB.

This work was conducted as part of a project to develop a core outcome set and thus was completed early and does not include more recent studies, however, the number of identified outcomes is unlikely to be significantly affected as there have been no radical developments during this time.

Most studies included in this review were judged to be at high risk of bias. This can partly be explained by the number of studies published prior to development and adoption of standardised reporting guidance for clinical studies. Although trial quality is important when performing a traditional systematic review, the data of interest for this work were the reported outcomes which are unlikely to be affected by study quality and thus has no implication on our results.

We intended to look at the quality of outcome reporting within studies, however the tool for assessment was extremely subjective. Ultimately, the quality of reporting did not affect our primary aim of identifying all previously reported outcomes and thus we abandoned this aspect of the work.

Whilst we might be criticised for excluding those studies of fibroids and adenomyosis that did not have a blood loss or quality of life primary outcome, we feel that this is justified as these studies focused on pain, pressure symptoms or volume reduction, factors that would not be relevant to all aetiologies underlying HMB.

### Interpretation

This systematic review has demonstrated that there is wide variation in the outcomes used in studies of HMB, however, by in depth review, it has also established that definitions, assessment tools and time-points for reporting outcomes are extremely varied. It was common to find that an outcome used by multiple studies was not defined in the same way, for example, ‘successful treatment’. We need to move away from these subjective outcomes and ensure standardised definitions.

Without standardisation of reporting, any attempt to synthesise or compare data is diluted by the inability to use all relevant studies. Thus, research is effectively wasted when it cannot contribute to the development of guidelines and impact clinical care. By developing a COS for HMB we will facilitate the use of all future research into this condition to influence clinical care, which will ensure value for money for funders, reduce the risk of selective outcome reporting, prevent research waste and ultimately allow decisions about patient care to be based upon maximum data.

Of note, these studies are evaluating HMB which is now more commonly diagnosed based upon a subjective definition (e.g. excessive menstrual blood loss which interferes with a woman’s physical, social, emotional and/or material quality of life (NICE, 2018)) than a quantitative one (e.g. more than 80ml blood loss). However, 44/49 reports of MBL as a primary outcome and 136/230 reports of MBL as a secondary outcome used either a PBLAC score or measured bleeding amount (alkaline haematin analysis). From the patient’s perspective, it is more important that they perceive their periods to be ‘normal’ or ‘better’ after treatment, rather than ‘statistically significantly reduced’ especially when the latter doesn’t necessarily translate to a clinically significant result.

Amenorrhoea is another outcome used commonly, however most treatments cannot hope to result in true amenorrhoea and thus using this as a primary outcome, for example in a trial of endometrial ablation, prevents use of that that data for comparison between treatments when developing clinical guidance. Again, definitions are varied- we identified eight different ways of assessing and reporting amenorrhoea in the 19 studies using it as a primary outcome. Amenorrhoea is an outcome for patients who specifically don’t want periods, rather than for those who just want ‘normal’ periods; continuing to call hysterectomy the ‘gold standard’ seems unfair to other effective treatments.

We removed ‘change’ outcomes (e.g., change in measured MBL, change in cycle length) during data analysis and kept the ‘endpoint’ version of outcomes (e.g., measured MBL, cycle length) as they are essentially the same outcome but with ‘change’ having methodological implications. ‘Change’ outcomes look at the change in something from baseline. It requires measurements to be taken at baseline and at follow-up. This has implications for study design and budget. Change studies often report the end point scores anyway.

Reporting time-points varied but with 3 and 12 months being most favoured. Standardising reporting time-points would allow more complete synthesis of outcome data and should be considered further when establishing outcome reporting guidance for clinical studies.

A recently published review of the variation of outcome reporting for adenomyosis identified that the most commonly reported outcomes were dysmenorrhoea, HMB and uterine volume (Tellum et al., 2021). Adenomyosis can be a cause of the symptom of HMB and thus it might be expected that a core outcome set for adenomyosis would have some overlap with the core outcome set for HMB. Not only would this be practical for researchers, but it might also provide some degree of ‘intra- rater’ validation, demonstrating that stakeholders identified the same important outcomes during the development process.

## Conclusion

The evident variation in reporting of outcomes in studies of HMB means that combination and comparison of study results is limited to those reporting similar outcomes and thus prohibits use of all the available evidence for specific treatments. Consequently, some data will not be used, and the time and money spent conducting the primary research will have been wasted as it cannot contribute towards evaluation of the treatment. We have demonstrated that a core outcome set is needed for studies of HMB, and we have subsequently carried out qualitative work with patients and an international consensus process involving all stakeholders to develop one. This core outcome set will be disseminated via publication in the coming months, and we hope that it will improve research and clinical care in this important area of women’s health.
